# Sex-specific differential survival of extra-pair and within-pair offspring in song sparrows, *Melospiza melodia*

**DOI:** 10.1098/rspb.2011.0173

**Published:** 2011-03-09

**Authors:** Rebecca J. Sardell, Peter Arcese, Lukas F. Keller, Jane M. Reid

**Affiliations:** 1 Institute of Biological and Environmental Sciences, School of Biological Sciences, Zoology Building, University of Aberdeen, Tillydrone Avenue, Aberdeen AB24 2TZ, UK; 2 Centre for Applied Conservation Research, Forest Sciences, 2424 Main Mall, University of British Columbia, Vancouver, CanadaV6T 1Z4; 3 Institute of Evolutionary Biology and Environmental Studies, University of Zurich, Winterthurerstrasse 190, 8057 Zurich, Switzerland

**Keywords:** extra-pair paternity, half-sibling, indirect benefits, polyandry

## Abstract

It is widely hypothesized that the evolution of female extra-pair reproduction in socially monogamous species reflects indirect genetic benefits to females. However, a critical prediction of this hypothesis, that extra-pair young (EPY) are fitter than within-pair young (WPY), has rarely been rigorously tested. We used 18 years of data from free-living song sparrows, *Melospiza melodia*, to test whether survival through major life-history stages differed between EPY and WPY maternal half-siblings. On average, survival of hatched chicks to independence from parental care and recruitment, and their total lifespan, did not differ significantly between EPY and WPY. However, EPY consistently tended to be less likely to survive, and recruited EPY survived for significantly fewer years than recruited WPY. Furthermore, the survival difference between EPY and WPY was sex-specific; female EPY were less likely to survive to independence and recruitment and lived fewer years than female WPY, whereas male EPY were similarly or slightly more likely to survive and to live more years than male WPY. These data indicate that extra-pair paternity may impose an indirect cost on females via their female offspring and that sex-specific genetic, environmental or maternal effects may shape extra-pair reproduction.

## 1. Introduction

Extra-pair mating, and multiple mating by a female within one reproductive cycle more generally, occurs in a wide range of organisms [1,2]. The evolution of such polyandrous behaviour, and resulting extra-pair paternity (EPP), may reflect both direct and indirect costs and benefits of EPP to males and females [2–5]. However, the magnitude and relative importance of these effects remain unclear [2,4–7]. Hypothesized direct benefits of extra-pair reproduction to females include fertility assurance, access to foraging areas, nest defence and future mate acquisition, but such benefits have received limited empirical support [2,3,6]. Consequently, indirect benefits, reflecting increased additive or non-additive genetic value of offspring sired by extra-pair males, are often hypothesized to be a primary force driving the evolution of female extra-pair reproduction [2,5,7–10]. Since understanding the adaptive function of EPP and multiple mating is central to understanding mating system evolution, accurate estimation of such indirect benefits remains a central aim in evolutionary ecology [8–10].

A critical prediction of the hypothesis that EPP reflects indirect genetic benefits to females is that extra-pair young (EPY) will be fitter than the within-pair young (WPY) they replace [2,5]. Numerous studies have tested this prediction by comparing traits between EPY and WPY. However, results are mixed; EPY can show higher [11,12], similar [13,14] or lower [15] trait values than WPY, varying both among traits and among studies that measured the same trait [5,7,16,17]. More importantly, most studies compare EPY and WPY with respect to juvenile traits that are assumed to predict fitness (such as offspring size, condition or immunocompetence), or microsatellite heterozygosity, rather than major fitness components or total fitness itself [7,18]. An obvious limitation of this approach is that offspring size, heterozygosity or physiological indices may not predict fitness. Observed differences in such traits may therefore provide misleading evidence of indirect benefits of EPP.

The ultimate but unachieved test for indirect benefits is to compare the total fitness of EPY and WPY [2,7,18]. However, it is also valuable to compare major fitness components such as survival, mating success and fecundity, thereby allowing the life history and selective processes underlying differences in overall fitness to be determined. Accordingly, several studies of socially monogamous but genetically polyandrous birds have tested whether EPY survive better than WPY (see the electronic supplementary material, table S1 for a literature review). Most such studies have measured offspring survival through early life-history stages (e.g. hatching to fledging or independence from parental care) because high levels of juvenile dispersal often prevent many offspring from being tracked [19–22] (see the electronic supplementary material, table S1). However, variation in individual fitness may substantially reflect variation in survival to recruitment and the total number of years survived (lifespan) [23,24]. Furthermore, genetic effects may be primarily manifested through adult traits such as lifespan or reproductive success [25]. Comparing recruitment and lifespan between EPY and WPY may therefore provide valuable insight into the potential indirect benefits of extra-pair reproduction.

It is increasingly suggested that genetic effects on fitness may depend on an individual's sex or the environment [25–27]. However, few studies comparing survival between EPY and WPY have explicitly tested for differential sex- or environment-specific effects (see the electronic supplementary material, table S1). Evidence of sex-specific differences in survival between EPY and WPY could suggest sexually antagonistic constraints on the evolution of extra-pair reproduction, analogous to those suggested to shape reproductive strategies more generally [28–32]. Likewise, environment-specific variation in the relative survival of WPY and EPY could suggest that the fitness consequences of extra-pair reproduction are context-dependent, as shown for female mating preferences [26,27]. Indeed, any increased fitness of EPY has been predicted to be primarily manifested under poor conditions, such as late in the breeding season [33–35]. Since such sex- or environment-specific variation may alter the magnitude of any overall indirect benefit of extra-pair reproduction [32,34,36], rigorous studies comparing fitness components between EPY and WPY should explicitly quantify sex- and environment-specific effects.

Precise comparison of fitness components between EPY and WPY requires not only rigorous measurement of these components but also appropriate control for variation owing to effects of natal and parental environments and maternal genes. One valuable approach is to directly compare EPY and WPY maternal half-siblings from the same brood or litter [2,11,18,35]. Accordingly, we used 18 years of data to compare the survival of maternal half-sibling EPY and WPY in free-living song sparrows, *Melospiza melodia*. We tested the specific hypotheses that EPY differ from their WPY half-siblings in their probability of survival from ringing (6 days post-hatch) to independence from parental care and recruitment, and in total lifespan. Furthermore, we tested whether differential survival of EPY versus WPY through these life-history stages differed between males and females or early and late season broods and hence showed differential sex- or environment-specific effects.

## 2. Methods

### (a) Study system

A small, resident population of socially monogamous song sparrows (numbering 33–131 adults during 1993–2010) inhabiting Mandarte Island, British Columbia, Canada, has been studied intensively since 1975 [37]. All song sparrows present on Mandarte have been individually colour-ringed as chicks or newly arrived immigrants, meaning that all individuals are identifiable by resighting. Both sexes can breed aged one and female song sparrows usually rear two broods per year (range 0–4) with median clutch size of four eggs (range 1–5) [37]. All territories were visited at least weekly during April–July each year to find all nests and identify both social parents (those defending the territory, incubating clutches and provisioning chicks). All nests were visited *ca* 6 days after hatching and all chicks were colour-ringed. Offspring reach independence from parental care *ca* 24–30 days post-hatch [37]. Territories and surrounding areas were therefore searched during this time to identify all surviving independent juveniles. All juveniles and adults surviving to subsequent breeding seasons were resighted with probability ≈1 [38]. Although there are several other islands nearby, immigration is infrequent (1.1 immigrants per year on average), but sufficient to maintain allelic diversity [37,39]. Local recruitment was 19.3 per cent of ringed chicks and 29.3 per cent of independent chicks during 1993–2009, which is high compared with other populations of song sparrows [37,40] and species with similar life histories [41]. Thorough searches of nearby islands have revealed few juvenile emigrants and no adults that have bred on Mandarte have ever been observed elsewhere [37,42–44]. Juvenile emigration is therefore likely to be relatively rare, and post-recruitment emigration is probably extremely rare [37,43]. Chick survival from ringing to independence was therefore estimated without any possible error owing to emigration, while survival to recruitment and total lifespan (the number of years an individual survived after ringing) were estimated with unusually high confidence (§4).

### (b) Paternity assignment and sexing

Each year during 1993–2009, a small blood sample was taken from virtually all ringed chicks, totalling 2343 of 2357 (99.4%) chicks from 854 broods, and virtually all adults. All sampled chicks were genotyped at 13 polymorphic microsatellite loci and assigned sires [45]. Virtually all ringed chicks were assigned as either WPY (sired by the male defending the female's territory during egg-laying) or EPY (sired by a different male) with high statistical confidence (≥95% at the individual level [45]). The maximum-likelihood probability of correctly excluding a female's social mate as sire was 0.9998 [45]. The estimated EPP rate was *ca* 28 per cent [45]. This is comparable to a nearby mainland population of song sparrows [46], and not remarkable for a passerine bird [2]. All chicks were sexed using the CHD-1 gene [47]. Molecular sexes were 100 per cent consistent with those attributed from reproductive behaviour for all recruited individuals.

### (c) Statistical analyses

Variation in survival probability may generally be best quantified using bespoke survival analyses which account for left-truncation [48]. However, such models are challenging to fit when random effects need to be included. We therefore used generalized linear mixed models to test whether each of three measures of survival through specific life-history stages differed between EPY and WPY maternal half-siblings: survival from ringing to independence from parental care, survival from ringing to recruitment and the total number of years survived from ringing (lifespan). The analysis of lifespan was further divided into two. The first analysis included all ringed chicks, providing a large sample size but possibly including some error owing to juvenile emigration (although this is probably small, see above). The second analysis was restricted to individuals that recruited, thereby eliminating any error owing to juvenile emigration but providing a smaller sample size. Since the two lifespan analyses were left-truncated to different degrees, effect sizes are not directly comparable.

All models included a chick's extra-pair status (EPY or WPY) as a fixed effect. Sex and season (i.e. whether a chick hatched in an early or late brood) have previously been shown to influence song sparrow survival and were therefore included as fixed effects [37]. Chicks from the first brood, each female raised to ringing each year were classified as early season broods, while chicks from all subsequent broods were classified as late season. This classification mapped tightly onto the observed bimodal distribution of laying dates and was therefore a biologically relevant definition for our dataset. Conclusions remained similar when analyses were rerun using Julian laying date rather than defining early and late season broods. All models included random effects of a chick's (or a recruit's) natal brood nested within social parent pair thereby accounting for variation both among broods raised by the same social parent pair and among broods raised by different social parent pairs. A random effect of cohort was also included in all models to account for known among-cohort variation in survival in song sparrows [37]. Two interactions, extra-pair status by sex and extra-pair status by season, were then modelled to test whether effects of extra-pair status on survival varied with sex or natal season. All main effects were retained in all models (even if not statistically significant across the current restricted dataset), owing to *a priori* knowledge of effects on survival. Interactions were removed if not significant. The magnitude and statistical significance of main effects were estimated from models without interactions.

Inbreeding coefficient (*f*) has sex-specific effects on survival on Mandarte [49]. However, *f* was not included in current analyses because EPY and WPY may differ in *f* if extra-pair reproduction allows inbreeding avoidance. Controlling for *f* may therefore control for part of the variation that our current aim is to measure. However, in practice, results remained quantitatively similar when analyses were rerun including *f* and a sex by *f* interaction.

All analyses were restricted to broods of known mixed paternity (where ≥1 EPY and ≥1 WPY survived to ringing), allowing comparison of survival between same-brood EPY and WPY half-siblings [18,21,50]. Broods that were not of known mixed paternity were excluded in case the occurrence of EPP covaries with female or pair quality, potentially biasing population-wide comparisons of EPY and WPY. The resulting sample size comprised 773 chicks from 245 broods and 177 social parent pairings for analyses of survival from ringing to independence and recruitment. Lifespan analyses were restricted to cohorts ringed during 1993–2003; all individuals from these cohorts were dead by 2010, meaning that the lifespans of all cohort members were known. The sample size for the chick lifespan analyses therefore comprised 471 chicks from 154 broods and 117 pairings. As there were few broods from which ≥1 EPY and ≥1 WPY recruited, analyses of recruit lifespan were also restricted to individuals from broods of known mixed paternity at ringing. The sample size for the recruit lifespan analyses was 99 recruits from 77 broods and 65 pairings. Although the total sample size of chicks was large, the number per cohort was relatively small (see the electronic supplementary material, table S2). We therefore did not test whether differences in survival between EPY and WPY differed among cohorts.

Data inspection suggested that the most appropriate error distribution to model lifespan was Poisson, although data were over-dispersed (see the electronic supplementary material, figures S1–S4). Lifespan models were consequently fitted using Markov Chain Monte Carlo (MCMC) (Bayesian) approaches assuming Poisson errors, additive overdispersion and log link to allow effects and associated uncertainty to be robustly estimated [51]. For consistency, Bayesian approaches were also used for analyses of survival from ringing to independence and recruitment (binary variables, using a logit link). Results were quantitatively similar when the binary models were fitted using maximum likelihood. For recruit lifespan analyses, the number of years survived was −1 transformed to meet Poisson assumptions. Analyses were run in R v. 2.11.1 using library MCMCglmm v. 2.06 [51,52]. Binary residual variance was fixed to 1 by convention. Priors on fixed effects were normally distributed, diffuse and proper with mean zero and large variance (10^8^). Priors on variance components were inverse-Wishart distributed with parameter *V* = 1 and degree of belief *n* = 0.002 [51]. Prior sensitivity analysis (and comparison with maximum-likelihood binary models) showed that results were robust to reasonable variation in these prior specifications (*V* = 0.1–1, *n* = 0.1–0.001). All models used burn-in 3000, 10 003 000 iterations and thinning interval 1000 to ensure autocorrelation among consecutive samples was low (less than 0.05). To assess statistical significance, 95% credible intervals surrounding posterior means were used. To aid visualization of biological effects, posterior means and credible intervals estimated on transformed scales were back-transformed to give estimated effect sizes on observed data scales marginalizing across random effects. Raw estimates of the proportion of chicks that survived from ringing to independence and recruitment, sex- and season-specific sample sizes and the distributions of chick and recruit lifespans, are provided in the electronic supplementary material, table S3 and figures S1–S4.

## 3. Results

### (a) Survival to independence

**Figure 1.**
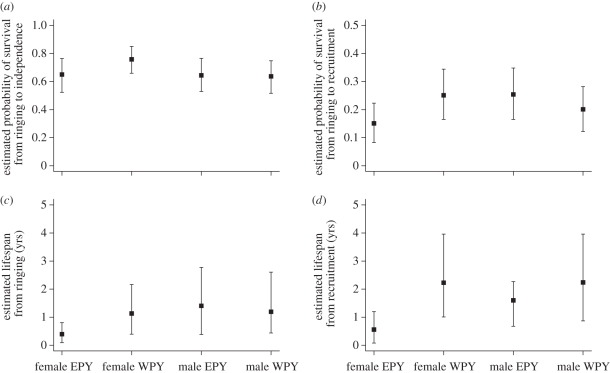
Back-transformed estimates (with 95% credible intervals) for (*a*) probability of survival from ringing to independence, (*b*) probability of survival from ringing to recruitment, (*c*) lifespan from ringing and (*d*) lifespan from recruitment for male and female extra-pair young (EPY) and within-pair young (WPY) from known mixed paternity broods.

The main effects of extra-pair status, sex and season on chick survival from ringing to independence were not significant (table 1). The extra-pair status by sex and extra-pair status by season interactions were also not significant (table 1). However, estimated absolute differences in survival showed that female EPY were on average *ca* 11 per cent less likely to survive than female WPY, and the 95 per cent credible interval for female WPY did not overlap the posterior mean for female EPY (figure 1). By contrast, male EPY were approximately as likely to survive as male WPY, and as female EPY (figure 1).

**Table 1.** Generalized linear mixed models explaining variation in (*a*) survival from ringing to independence, (*b*) survival from ringing to recruitment, (*c*) lifespan from ringing and (*d*) lifespan from recruitment. (Each model was run (i) including main effects only and (ii) including interaction terms. Sample sizes (number of chicks/recruits and mixed paternity broods), posterior means, 95% credible intervals (95% CI) and MCMC *p*-values are presented. Estimates for the extra-pair status by season interactions are from models including this term. All other estimates are from models excluding this term. Intercepts represent female within-pair young from early broods. Bold indicates statistically significant effects.)

	model	sample size		estimate	intercept	extra-pair status	sex	season	extra-pair status by sex	extra-pair status by season
(*a*)	independence	773 chicks, 245 broods	(i)	mean 95% CI	**1.18 (0.59, 1.77**)	−0.28 (−0.68, 0.12)	−0.36 (−0.78, 0.07)	0.19 (−0.31, 0.71)	—	—
				MCMCp	**0.01**	0.19	0.09	0.45	—	—
			(ii)	mean 95% CI	**1.34 (0.75, 1.98**)	**−0.61 (−1.20, −0.01**)	**−0.68 (−1.27, −0.09**)	0.19 (−0.33, 0.69)	0.64 (−0.24, 1.46)	−0.22 (−1.05, 0.59)
				MCMCp	**0.01**	**0.04**	**0.02**	0.47	0.13	0.60
(*b*)	recruitment	773 chicks, 245 broods	(i)	mean 95% CI	**−1.51 (−2.10, −0.96**)	−0.17 (−0.58, 0.27)	0.15 (−0.26, 0.60)	−0.44 (−0.89, 0.00)	—	—
				MCMCp	**0.01**	0.44	0.47	0.05	—	—
			(ii)	mean 95% CI	**−1.28 (−1.88, −0.74**)	**−0.76 (−1.38, −0.11**)	−0.34 (−0.93, 0.24)	**−0.46 (−0.92, −0.02**)	**1.11 (0.22, 1.99**)	−0.55 (−1.42, 0.33)
				MCMCp	**0.01**	**0.02**	0.26	**0.04**	**0.01**	0.22
(*c*)	chick lifespan	471 chicks, 154 broods	(i)	mean 95% CI	**−2.32 (−3.14, −1.52**)	−0.38 (−0.96, 0.16)	**0.60 (0.01, 1.16**)	−0.34 (−0.94, 0.27)	—	—
				MCMCp	**0.01**	0.18	**0.04**	0.27	—	—
			(ii)	mean 95% CI	**−2.04 (−2.83, −1.23**)	**−1.09 (−1.95, −0.15**)	0.06 (−0.72, 0.81)	−0.36 (−0.97, 0.21)	**1.23 (0.09, 2.45**)	−0.54 (−1.73, 0.61)
				MCMCp	**0.01**	**0.02**	0.88	0.23	**0.04**	0.36
(*d*)	recruit lifespan	99 recruits, 77 broods	(i)	mean 95% CI	−0.07 (−0.70, 0.53)	**−0.69 (−1.36, −0.04**)	0.34 (−0.33, 0.98)	−0.14 (−0.76, 0.52)	—	—
				MCMCp	0.85	**0.04**	0.31	0.67	—	—
			(ii)	mean 95% CI	0.12 (−0.54, 0.73)	**−1.48 (−2.70, −0.30**)	−0.01 (−0.82, 0.77)	−0.16 (−0.83, 0.46)	1.16 (−0.27, 2.60)	0.32 (−0.97, 1.66)
				MCMCp	0.68	**0.01**	0.99	0.62	0.11	0.64

### (b) Survival to recruitment

The main effects of extra-pair status and sex on chick survival from ringing to recruitment were not significant (table 1). The main effect of season was marginally non-significant; chicks hatched in late broods tended to be less likely to recruit than chicks hatched in early broods (table 1). The extra-pair status by sex interaction was significant; female EPY were less likely to recruit than female WPY, whereas male EPY were slightly more likely to recruit than male WPY (table 1 and figure 1). Estimated absolute differences in survival showed that on average, female EPY were *ca* 10 per cent less likely to recruit than female WPY, while male EPY were *ca* 5 per cent more likely to recruit than male WPY. The extra-pair status by season interaction was not significant (table 1).

### (c) Chick lifespan

The main effects of extra-pair status and season on chick lifespan from ringing were not significant, but lifespan varied significantly with sex; males survived more years than females (table 1). However, the extra-pair status by sex interaction was also significant; female EPY survived fewer years than female WPY, whereas male EPY tended to survive more years than male WPY (figure 1). Estimated absolute differences in survival showed that on average, female EPY survived *ca* 0.7 fewer years than female WPY, while male EPY survived *ca* 0.2 more years than male WPY. The extra-pair status by season interaction was not significant (table 1).

### (d) Recruit lifespan

The main effect of extra-pair status on the lifespan of recruits was significant; EPY survived fewer years on average than WPY (table 1 and figure 1). The main effects of sex and season were not significant, nor were the extra-pair status by sex and extra-pair status by season interactions (table 1 and figure 1). Estimated absolute differences in survival showed that on average, recruited female EPY lived *ca* 1.7 fewer years than recruited female WPY, and recruited male EPY lived *ca* 0.6 fewer years than recruited male WPY.

## 4. Discussion

A testable prediction of the hypothesis that extra-pair reproduction partly reflects indirect genetic benefits to females is that offspring sired by extra-pair males will be fitter than their half-siblings that were sired by a female's social mate [2,5,35]. We used comprehensive data from a resident population of song sparrows with high natal and breeding philopatry to test whether survival through major life-history stages differed between extra-pair and within-pair maternal half-siblings, and whether these effects depended on offspring sex or natal season.

### (a) Overall effects of extra-pair status

On average, the survival of EPY from ringing to independence from parental care and recruitment, and their total lifespan, did not differ significantly from the survival or lifespan of WPY. However, EPY tended to survive less well than WPY through these stages, and recruited EPY lived fewer years than recruited WPY. The trend for lower survival in EPY was therefore consistent across these life-history stages. Since emigration is absent prior to independence and probably rare subsequently (§2), these patterns most probably reflect a tendency for lower true survival in EPY rather than greater emigration.

Although indirect genetic benefits are often suggested to be one main force driving female extra-pair reproduction, some previous studies also found that EPY tend to have lower survival than WPY [5]. Indeed, two recent meta-analyses concluded that there is little overall evidence that indirect genetic benefits drive female extra-pair reproduction [5,7]. The tendency towards lower average survival of EPY in our study suggests that EPP may not provide an indirect fitness benefit for female song sparrows. Moreover, because reproductive lifespan is a major determinant of fitness in song sparrows and other species [23,37], the shorter lifespan of recruited EPY compared with recruited WPY suggests that EPP may even impose an indirect fitness cost on females. However, since survival may trade off against reproductive success [25,26], EPP could still provide an indirect genetic benefit to females if EPY have substantially higher reproductive success than WPY, or if their own offspring are fitter. Comparison of EPY and WPY in terms of their lifetime number of offspring and grandoffspring, and in pre-ringing survival, is therefore still required [2,5,7,18]. In the absence of sufficient compensation through reproductive success or survival to ringing, the low survival and short lifespans of EPY compared with their half-sibling WPY suggest that EPP may reflect direct rather than indirect benefits to females or be predominantly male-driven (reflecting sexual conflict) [4,5,7].

### (b) Sex-specific effects

Although the overall tendency for EPY to have lower mean survival than WPY suggests that EPP might impose an indirect cost on females, relationships between extra-pair status and survival differed between male and female offspring, indicating a more complex situation. Moreover, estimated biological effects were substantial. Female EPY were *ca* 14 and 40 per cent less likely to survive from ringing to independence and recruitment relative to female WPY, and had *ca* 65 per cent shorter lifespans. Furthermore, recruited female EPY lived *ca* 75 per cent fewer years relative to recruited female WPY. By contrast, male EPY and male WPY were approximately equally likely to survive from ringing to independence, while male EPY were *ca* 21 per cent more likely to survive from ringing to recruitment and lived *ca* 15 per cent more years relative to male WPY (although these effects were not in themselves statistically significant). Once recruited, however, male EPY lived *ca* 29 per cent fewer years relative to male WPY. Overall, these results demonstrate substantial sex-specific effects on the differential survival of EPY versus WPY, driven predominantly by the considerably lower survival of female EPY.

This sex-specific differential survival of EPY versus their WPY maternal half-siblings could reflect various different mechanisms. The observed patterns could conceivably reflect sex-biased emigration with respect to extra-pair status if female EPY were more likely to emigrate than female WPY, but male EPY were no more likely to emigrate than male WPY. However, the survival difference between female EPY and WPY occurred to some degree across all life-history stages, including those that cannot have been affected by emigration (i.e. survival from ringing to independence), or are very unlikely to have been affected (i.e. recruit lifespan). Estimated sex-specific differences in apparent survival of EPY versus WPY, therefore, most likely reflect differences in true survival.

Sex-specific differential survival of EPY versus WPY could potentially reflect differences in environmental or maternal effects between EPY and WPY that differentially affect the survival of males and females. Indeed, the interpretation of any maternal half-sibling comparison as demonstrating indirect genetic benefits of extra-pair reproduction assumes that any environmental or parental effects do not differ between EPY and WPY. In fact, recent evidence shows that EPY may be laid early within clutches, and that observed phenotypic superiority of EPY over WPY can consequently be environmental and/or maternal rather than genetic [50,53]. Furthermore, females may be predicted to modify investment in eggs depending upon their paternity or mate attractiveness [54], and maternal investment may also be sex-specific and interact with laying order to affect offspring growth and survival [55,56]. The possibility that such mechanisms may underlie observed variation in offspring survival in song sparrows remains to be tested. However, our observation that female EPY survived poorly cannot easily be explained by EPY hatching early within a brood, as has been observed elsewhere [50,53].

Sex-specific differential survival of EPY versus WPY could also reflect sex-specific effects of inbreeding if mean *f* differs between EPY and WPY and *f* affects male and female survival differently. Indeed, EPY would have lower *f* than WPY on average if extra-pair reproduction reflects inbreeding avoidance, as is widely hypothesized [8,10]. This hypothesis remains to be explicitly tested in our system. However, although inbreeding depression in adult survival is sex-specific in song sparrows, inbreeding depression in juvenile survival to recruitment is not [49]. Thus, it appears unlikely that differential inbreeding depression could cause the observed variation in offspring survival; this would require females to produce EPY daughters but not EPY sons that are more inbred than their WPY of the same sex. Moreover, our results remained similar after controlling for variation in offspring *f* (§2).

A further possibility is that sex-specific differential survival of EPY versus WPY may reflect sexually antagonistic genetic effects on offspring survival. Recent studies suggest that sexually antagonistic effects may be common in a range of taxa, for example, causing males with high fitness or mating success to produce sons with high fitness but daughters with low fitness [29,30,32,36]. Our results show that successful extra-pair sires produce daughters that survive poorly but sons that survive at least averagely well, and therefore mirror this general pattern. Because our comparison between EPY and WPY was purely phenotypic, we cannot explicitly test whether the observed sex-specific differential survival may reflect sexually antagonistic genetic effects. Nonetheless, if the observed patterns did reflect such effects, the potential for extra-pair reproduction to evolve through indirect genetic benefits may be limited [32,36], but not entirely precluded if the total fitness benefits of producing extra-pair sons outweigh the costs of producing extra-pair daughters [25]. Indeed, by biasing the sex ratio of EPY towards males [57], females could maximize the fitness benefit of EPP. However, for EPY and WPY hatchlings in mixed paternity song sparrow broods on Mandarte, the sex ratios (proportion of males) were 0.52 and 0.49, respectively. These proportions do not differ significantly from 50 : 50 (exact binomial tests, *p* = 0.52, 0.62) or from each other (Fisher's exact test, *p* = 0.38). There is therefore no evidence that female song sparrows manipulated the sex of EPY. The fitness benefit of producing male EPY would therefore need to be large to compensate for the fitness cost of producing female EPY and provide an overall indirect benefit of EPP to females. Instead, the similar posterior means and considerable overlap of the credible intervals for survival of male WPY and EPY suggested that any fitness benefit from male EPY offspring is likely to be small. However, the hypotheses that sexually antagonistic effects may underlie the sex-specific differential survival of EPY versus WPY, or drive female extra-pair reproduction overall, remain to be definitively tested.

Despite increasing general interest in sex-specific variation in fitness, only one previous study comparing survival between EPY and WPY reported an explicit test for sex-specific effects [18] (see the electronic supplementary material, table S1). In coal tits (*Periparus ater*) recruit lifespan did not differ significantly among male and female EPY and WPY [18]. Sex-specific differential lifespans of recruited EPY and WPY were not evident in our study either; both male and female EPY recruits lived fewer years than WPY. Instead, we observed sex-specific differential survival of EPY and WPY among half-sibling chicks. The absence of other studies reporting a sex-specific difference in the relative fitness of EPY and WPY may therefore reflect both a lack of studies that test for sex-specific effects and also the choice of traits and life-history stages used to estimate fitness. Our data show that measuring survival from hatching to recruitment and beyond may be essential to accurately quantify sex-specific fitness effects of extra-pair status.

### (c) Seasonal effects

Survival from ringing to recruitment and beyond tended to be lower for chicks hatched later in the season (as previously observed in song sparrows [37]). However, the relative survival of EPY and WPY and therefore the fitness consequences of EPP for females did not vary with season. By contrast, the only other study system where differential survival of EPY and WPY in early versus late broods was estimated showed that coal tit EPY had higher recruitment if hatched late in the season but tended to have lower recruitment if hatched early in the season, with no average effect of extra-pair status across all broods [34]. If anything, EPY on Mandarte tended to be less likely to survive from ringing to recruitment if hatched late in the season (table 1). The prediction that EPY should have higher fitness under poorer conditions [33–35] was therefore not supported with respect to hatch season.

### (d) Conclusion

Overall, we show that the effect of extra-pair status on survival through major life-history stages is sex-specific in song sparrows; female EPY had lower survival than female WPY, while male EPY had similar or slightly higher survival than male WPY. Explicitly quantifying the relative survival of male and female EPY and WPY may therefore be essential to understand the indirect fitness consequences of extra-pair reproduction. Whether the observed sex-specific differential survival of EPY versus WPY is mirrored in reproductive success and therefore total fitness remains to be investigated. If it is, and EPY have lower fitness than WPY on average, then extra-pair reproduction may result in an indirect fitness cost to females via their female extra-pair offspring. Other hypotheses for the evolution of polyandry in socially monogamous species, such as sexual conflict [4,5,7] would then require robust testing.
